# Genome-Wide Identification of *CYP75* Gene Family in *Rhododendron simsii* and Functional Analysis of Its Role in Promoting Anthocyanin Biosynthesis

**DOI:** 10.3390/plants15101472

**Published:** 2026-05-12

**Authors:** Yu-Hang Jiang, Yong-Hong Jia, Ze-Hang Wu, Gao-Yuan Hu, Bin-Ying Sun, Chen-Xin Xie, Qing-Hao Wang, Chao Yu, Hai-Chao Hu, Xiao-Hong Xie, Yue-Yan Wu

**Affiliations:** 1 College of Biological & Environmental Sciences, Zhejiang Wanli University, Ningbo 315100, China; 2023881083@zwu.edu.cn (Y.-H.J.); wzh881018@163.com (Z.-H.W.); gaoyuan.hu@163.com (G.-Y.H.); 18668290372@163.com (B.-Y.S.); 19857040192@163.com (C.-X.X.); yuchao13@zwu.edu.cn (C.Y.); huhdfs@163.com (H.-C.H.); zwuxxh@zwu.edu.cn (X.-H.X.); wyy2000@zwu.edu.cn (Y.-Y.W.); 2 School of Life Sciences, Hebei University, Baoding 071002, China; wqh15657811121@163.com

**Keywords:** *Rhododendron simsii*, *Rhododendron × hybridum*, *CYP75* gene family, *RhF3′5′H*, functional analysis

## Abstract

The flower color of *Rhododendron* is primarily determined by anthocyanin biosynthesis, with cytochrome P450 CYP75 family members, particularly flavonoid 3′,5′-hydroxylase (F3′5′H), playing a central role. However, the composition and functional characterization of *CYP75* genes in *Rhododendron* remain insufficiently explored. This study performed genome-wide identification of the CYP75 gene family using the *Rhododendron simsii* reference genome and functionally characterized the corresponding F3′5′H homolog cloned from *Rhododendron × hybridum* petals (red cultivar and pink cultivar). Seven *RsCYP75* genes were identified, categorized into two subfamilies: *RsCYP75A* (A1–A5) and *RsCYP75B* (B1–B2), with a prominent cluster on chromosome 13. All encoded proteins contained a conserved cytochrome P450 domain and typical heme-binding motifs. Among these, *RhCYP75A2* showed the highest expression level in red petals at full blooming period and was designated as *RhF3′5′H*. *RhF3′5′H* encodes a basic membrane protein with the characteristic *F3′5′H* motif, with its transcript most abundant in flowers. Transient overexpression of *RhF3′5′H* in red *R. × hybridum* petals resulted in a 9.74-fold increase in its transcript levels and a 1.25-fold increase in anthocyanin content compared to that in the control accompanied by the up-regulation of *CHS*, *F3H*, *DFR* and *ANS*. Conversely, *RhF3′5′H* silencing reduced anthocyanin accumulation but increased *CHS* and *F3H* transcript levels, suggesting a compensatory transcriptional response in the upstream anthocyanin pathway. Moreover, RhF3′5′H was heterologously expressed in *E. coli* Rosetta as an MBP fusion protein, purified, and identified by LC-MS/MS and ELISA. The protein showed the ability to promote anthocyanin accumulation. Molecular docking analysis demonstrated that RhF3′5′H can bind to naringenin and dihydrokaempferol. These results confirm that RhF3′5′H is a functional F3′5′H-type CYP75A enzyme and a positive regulator of anthocyanin accumulation in *Rhododendron* petals. This work enriches the *CYP75* gene catalog in *Rhododendron* and provides candidate genes for future studies on flower color regulation and molecular breeding.

## 1. Introduction

Flower color is one of the key traits determining the ornamental and economic value of plants and directly influences their significance in horticultural and ecological applications [1]. Anthocyanins, a class of water-soluble pigments, are widely distributed in petals, fruits and leaves, and represent one of the major determinants of floral coloration [2]. The accumulation of anthocyanins underlies the manifestation of red, purple and blue hues in plants and also contributes to antioxidant capacity, protection against ultraviolet radiation, and resistance to pathogens and pests [3]. Anthocyanin biosynthesis is orchestrated by a series of structural genes in the flavonoid pathway, including *CHS*, *CHI*, *F3H*, *F3′H*, *F3′5′H*, *DFR*, *FLS*, *ANS*, and *UFGT*, which together determine the synthesis and accumulation of anthocyanins [4,5].

Flavonoid 3′,5′-hydroxylase (F3′5′H) plays a pivotal role in this pathway by catalyzing the 3′,5′-hydroxylation of dihydrokaempferol (*DHK*) to form dihydromyricetin (*DHM*), a key precursor of delphinidin-derived anthocyanins that are closely associated with blue or purple floral pigmentation [6,7]. In contrast, flavonoid 3′-hydroxylase (F3′H) redirects metabolic flux toward cyanidin-derived anthocyanins, thereby contributing to red or pink pigmentation in flowers [7,8]. Consequently, the metabolic competition between F3′5′H and F3′H largely determines the final balance between blue- and red-type anthocyanin accumulation and thus the resultant flower color [9]. Although *F3′5′H* genes have been extensively investigated in several plant species—particularly in *Petunia hybrida* [10], and *barley* [11], where overexpression or silencing of *F3′5′H* has been shown to modulate anthocyanin biosynthesis—our understanding of *F3′5′H* function in many horticultural taxa remains incomplete. In particular, its role in *Rhododendron* is still poorly defined, despite the genus being an important ornamental group with remarkable floral color diversity and anthocyanin-rich petals whose regulatory mechanisms are not yet fully clarified [12]. Clarifying the function of *F3′5′H* in *Rhododendron* is therefore a primary objective of this study. Flavonoid 3′,5′-hydroxylases belong to the CYP75 subfamily of the cytochrome P450 superfamily, members of which show sequence diversity and functional variation across plant lineages [13]. Members of this gene family play important roles in multiple plant species; for example, *CYP75* genes participate in the biosynthesis of anthocyanins and other secondary metabolites in *Scutellaria baicalensis* [14] and *Lagerstroemia indica* [15]. Comparative genomic evidence further indicates that the *CYP75* gene family has undergone pronounced gene expansion and functional diversification across different lineages. In particular, lineage-specific duplications have been documented in *Orchidaceae* (e.g., *Cymbidium goeringii)* [13] and *blueberry* (*Vaccinium corymbosum*) [16], where expanded CYP75A (*F3′5′H*) and CYP75B (*F3′H*) subfamilies have evolved specialized roles in flavonoid hydroxylation and secondary metabolite production. Despite significant progress in understanding anthocyanin biosynthesis in many plant species, the molecular mechanisms underlying flower color regulation in *Rhododendron*, a genus with remarkable floral color diversity, remain poorly understood. While previous studies have identified key genes involved in anthocyanin biosynthesis, such as chalcone synthase (*CHS*), flavonoid 3′-hydroxylase (*F3′H*), the specific roles of the *CYP75* gene family members in regulating flower color in *Rhododendron* have not been systematically characterized. Furthermore, the interaction between these genes and the complex regulatory networks that determine flower pigmentation is still unclear.

This study employs whole-genome sequencing and gene family analysis to identify and systematically characterize the members of the *CYP75* gene family in *Rhododendron simsii*, with a particular focus on the function of the *F3′5′H* gene. The aim is to explore, through genomic and molecular biology approaches, the role of F3′5′H in *Rhododendron* and its involvement in flower color formation. Using *Rhododendron × hybridum* as the study material, we performed cloning and expression analyses of the *RhF3′5′H* gene, followed by functional assays including transient overexpression and gene-silencing analyses in petals. This design was adopted because *R. simsii* represents a key ancestral genomic resource for cultivated *Rhododendron*, whereas *R. × hybridum* provides experimentally tractable horticultural materials for petal-based functional assays. In parallel, the transcript levels of key structural genes in the anthocyanin biosynthetic pathway (*CHS, F3H, DFR* and *ANS*) were quantified by qPCR under both overexpression and silencing conditions to assess pathway-level transcriptional responses. These experiments were designed to clarify the regulatory role of RhF3′5′H in anthocyanin accumulation and to reveal potential feedback/compensatory regulation in the upstream pathway. Furthermore, this study provides an initial characterization of the *CYP75* gene family in *Rhododendron*, offering preliminary insights into family structure, phylogenetic relationships, and expression features, while functionally examining the role of *RhF3′5′H* in anthocyanin accumulation and flower-color regulation.

## 2. Result and Analysis

### 2.1. Gene Structure and Chromosomal Distribution of CYP75

**Figure 1:**
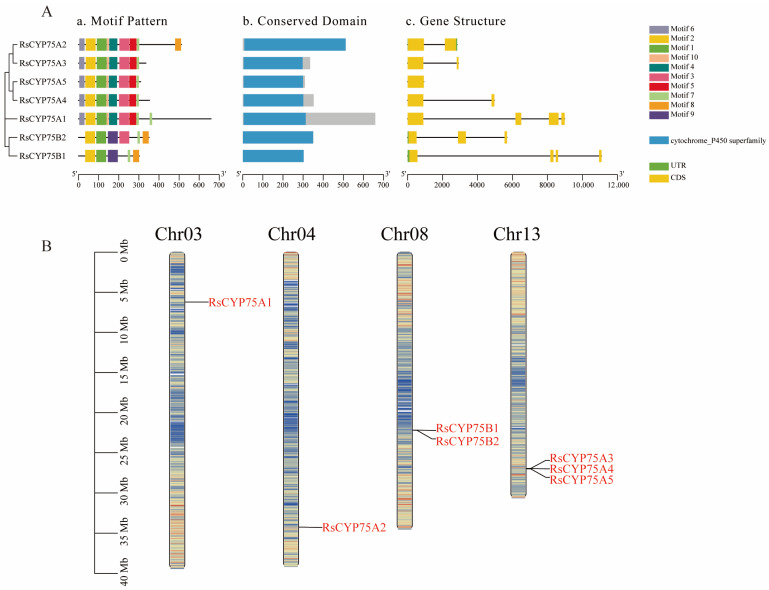
Structural characteristics and chromosomal distribution of the RsCYP75 gene family in *Rhododendron simsii*. (**A**) Structural features of RsCYP75 family members, including (**a**) motif pattern, (**b**) conserved domain organization, and (**c**) gene structure. In panel (**a**), different colored boxes represent distinct conserved motifs identified in the RsCYP75 proteins. In panel (**b**), blue boxes indicate the annotated cytochrome P450 superfamily domain, whereas gray regions represent non-conserved regions outside the core annotated domain. In panel (**c**), yellow boxes indicate coding sequences (CDS), green boxes indicate untranslated regions (UTRs), and black lines represent introns. (**B**) Chromosomal distribution of the seven RsCYP75 genes on chromosomes Chr03, Chr04, Chr08, and Chr13 of the *R. simsii* genome. The chromosome ideograms are shown with megabase (Mb) scale on the left, and red labels indicate the physical positions of individual *RsCYP75* genes.

Seven *RsCYP75* genes identified in the *Rhododendron simsii* genome were analyzed for motif composition, conserved domain organization, gene structure, and chromosomal distribution (Figure 1). Based on their phylogenetic relationships and motif composition, the identified genes were classified into two subfamilies, namely RsCYP75A (RsCYP75A1–RsCYP75A5) and RsCYP75B (RsCYP75B1–RsCYP75B2). Conserved domain analysis revealed that all RsCYP75 proteins contained the typical cytochrome P450 superfamily domain, suggesting that the core functional region of this family is highly conserved. However, differences were observed in the non-conserved regions outside the core domain, particularly in RsCYP75A1. Gene structure analysis further showed obvious variation in gene length and CDS organization among family members, implying structural diversification during evolution. Chromosomal mapping demonstrated that the seven RsCYP75 genes were unevenly distributed on four chromosomes, with three genes located on Chr13, two on Chr08, and one each on Chr03 and Chr04. The clustering of multiple RsCYP75 genes on Chr13 suggests that local duplication events may have contributed to the expansion of this gene family.

### 2.2. Evolutionary Analysis of CYP75

**Figure 2:**
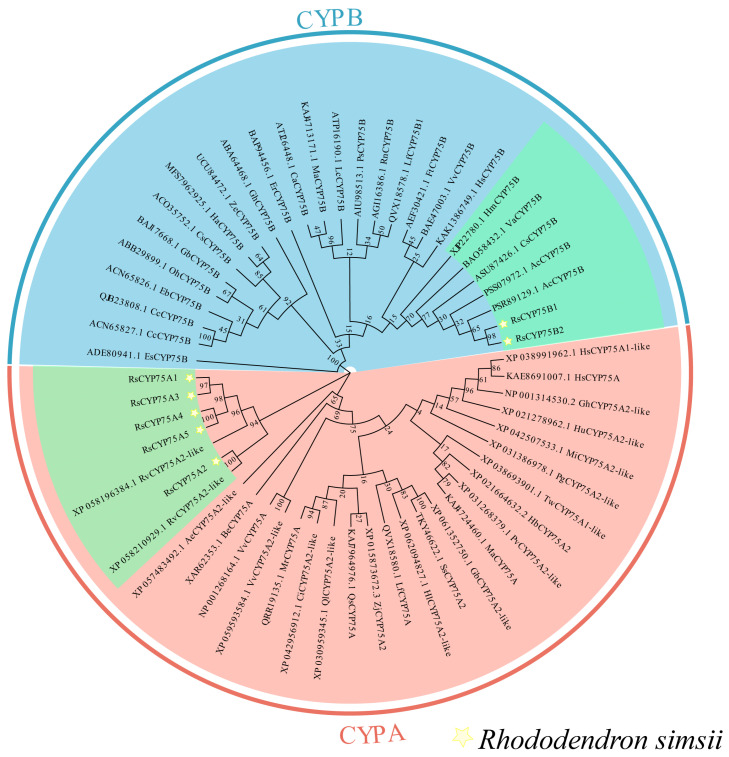
Phylogenetic analysis of RsCYP75 proteins from *Rhododendron simsii* and representative CYP75 proteins from other plant species. The blue clade represents the CYP75B subfamily, whereas the pink clade represents the CYP75A subfamily. Stars indicate the RsCYP75 proteins identified in this study.

Phylogenetic analysis showed that the RsCYP75A subfamily clustered most closely with CYP75 homologs from *Rhododendron vialii*, whereas the RsCYP75B subfamily was most closely related to *Actinidia chinensis* CYP75B (PSR89129.1) (Figure 2).

### 2.3. Cis-Acting Element Analysis of CYP75 Members in Rhododendron simsii

**Figure 3:**
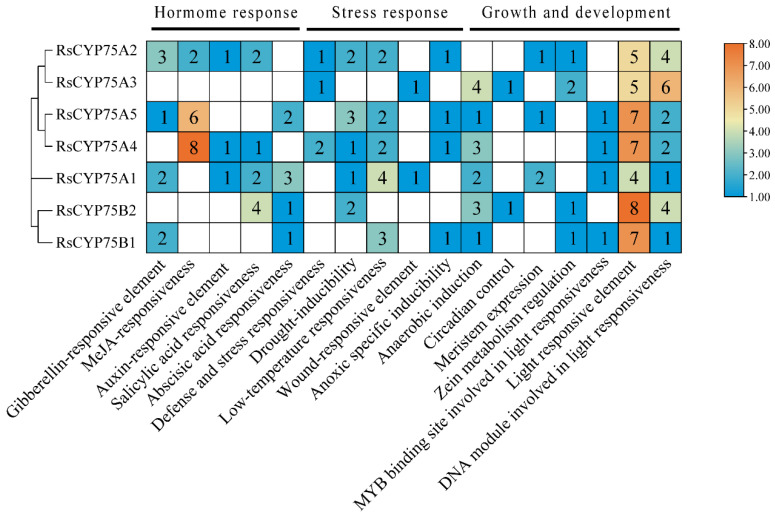
Identification of cis-acting elements in the *RsCYP75* promoter. Left phylogenetic tree: Vertical dendrogram branches represent the evolutionary relationships of the *RsCYP75* gene; Right numeric (1–8) + color dual-coding association strength (right color scale bar “blue→orange” corresponds to values “low→high,” with blue indicating weak association and orange indicating strong association).

The putative promoter regions (2000 bp upstream of the start codon) of *RsCYP75* genes were analyzed to investigate their regulatory features, revealing a total of 17 types of cis-acting elements. These elements were primarily associated with hormone responsiveness, stress responses, and growth and development. Notable differences were observed in the distribution of hormone-, stress-, and development-related cis-elements between the promoters of *RsCYP75A* and *RsCYP75B* subfamily members. Specifically, *RsCYP75A* promoters contained a broader range of hormone-responsive elements, particularly those related to MeJA, as well as a higher abundance of elements linked to stress responses. In contrast, the promoters of *RsCYP75B* genes were more enriched in elements associated with growth and developmental processes (Figure 3).

### 2.4. Analysis of the Physicochemical Properties and Protein Structure of RsCYP75

In the current study, the number of amino acids encoded by *RsCYP75* members, determined using ExPASy (https://web.expasy.org/protparam/, accessed on 17 July 2025), ranged from 303 to 659, with their molecular weights varying from 33.35 to 72.53 kDa. Most of these members had an isoelectric point (pI) greater than 7, while only *RsCYP75A1* had a pI of 6. Predictions of the grand average of hydropathicity (GRAVY) showed that the RsCYP75 proteins displayed slight variation in hydropathicity, with four members showing negative GRAVY values and three members showing weakly positive values (Supplementary Table S2).

**Figure 4:**
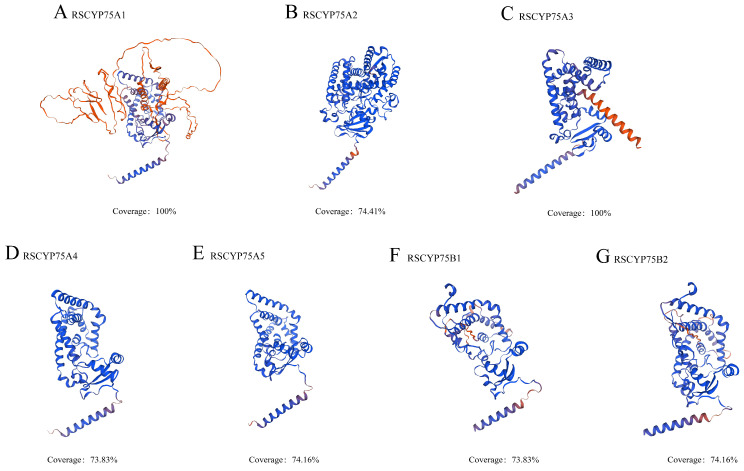
Three-dimensional structural analysis of RsCYP75 proteins. Predicted tertiary structures of the seven RsCYP75 proteins, including RsCYP75A1 (**A**), RsCYP75A2 (**B**), RsCYP75A3 (**C**), RsCYP75A4 (**D**), RsCYP75A5 (**E**), RsCYP75B1 (**F**), and RsCYP75B2 (**G**), generated using AlphaFold2. The RsCYP75 proteins displayed a relatively conserved overall spatial conformation, while certain members showed local structural variation. The coverage value presented below each model represents the percentage of the protein sequence included in the predicted three-dimensional structure.

The tertiary structure prediction revealed that members of the RsCYP75 family possess a highly conserved CYP450 domain. Specifically, the proteins encoded by the CYP75A subfamily genes contain three conserved sequences: the binding domain of the enzyme’s globular region, the oxygen molecule-binding domain, and the heme-binding domain (Figure 4).

### 2.5. Flower-Enriched Expression and Subcellular Localization of RhF3′5′H

**Figure 5:**
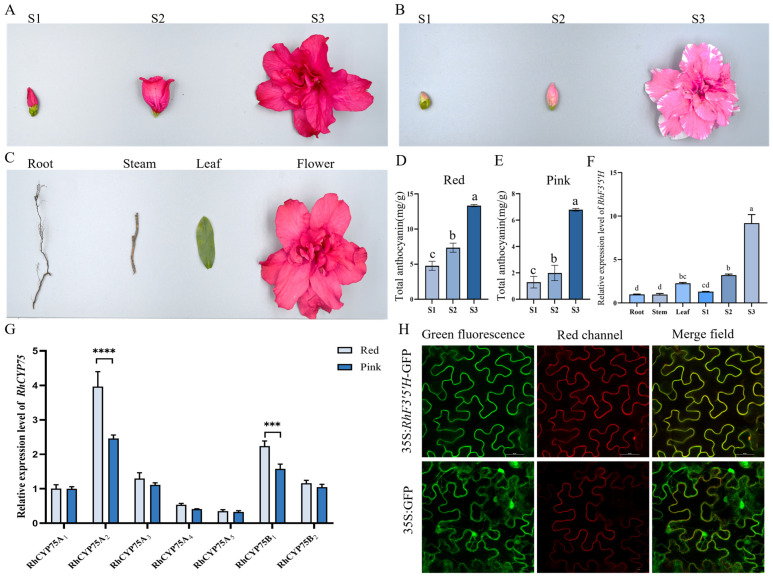
Flower-enriched expression of F3′5′H, gene expression analysis, subcellular localization, and measurement of total anthocyanin content in red and pink *Rhododendron × hybridum* flowers. (**A**) Red *Rhododendron × hybridum* flowers at the S1–S3 stages. (**B**) Pink *Rhododendron × hybridum* flowers at the S1–S3 stages. (**C**) Different tissues of red *Rhododendron × hybridum*. (**D**) Measurement of total anthocyanin content in red *Rhododendron × hybridum* flowers at the S1-S3 stages. (**E**) Measurement of total anthocyanin content in pink *Rhododendron × hybridum* flowers at the S1-S3 stages. (**F**) Relative expression level of *RhF3′5′H* in different tissues/developmental stages of *Rhododendron × hybridum*. Data are presented as mean ± SD (n = 3). Different lowercase letters above the bars indicate significant differences among groups, as determined by one-way ANOVA followed by Tukey’s multiple-comparison test (*p* < 0.05); (**G**) Relative expression of *RhCYP75* in red and pink cultivars, as indicated on the x-axis. Differences between the two cultivars were analyzed using Student’s *t*-test. Data are shown as mean ± SD (n = 3). *** *p* < 0.001, **** *p* < 0.0001. (**H**) Subcellular localization of *RhF3′5′H* in *Nicotiana benthamiana* epidermal cells. Green fluorescence represents the RhF3′5′H–GFP signal, red fluorescence indicates the CDBK marker, and the merged images show the co-localization of the two signals. Yellow fluorescence in the merged images indicates that RhF3′5′H is predominantly localized to the plasma membrane. Scale bars = 50 μm.

Sequence alignment identified seven homologous genes in *Rhododendron × hybridum*, all of which were found to belong to the RhCYP75 family. To investigate the expression levels of CYP75 family members in *Rhododendron × hybridum*, the petal color parameters of red and pink cultivars at full bloom were measured using the Royal Horticultural Society Colour Chart (RHSCC). The a* values were significantly higher in red flowers compared to pink flowers, as detailed in Supplementary Table S3. Additionally, total anthocyanin content was measured in the petals of red and pink cultivars at the S1–S3 stages (Figure 5D,E). The results revealed that the highest anthocyanin content was found in the red *Rhododendron × hybridum* petals at the full-bloom stage (S3) (Figure 5C,F). Based on these findings, qRT-PCR was employed to analyze the expression of *RhCYP75A2* in various tissues of red *Rhododendron × hybridum*. *RhCYP75A2* is highest in both red and pink *Rhododendron × hybridum* flowers at full bloom (S3) (Figure 5A,B,G). Furthermore, *RhCYP75A2* belongs to the CYP75A subfamily and is therefore named *RhF3′5′H*. Gene expression analysis across different tissues showed that RhCYP75A2 was expressed in multiple tissues but was most highly expressed in flowers, particularly at the full-bloom stage (S3).

Sequence alignment showed that members of the CYP75 family, especially the CYP75A subfamily, exhibit high homology with the F3′5′H protein identified in *Petunia* [17]. Sequence analysis of RhF3′5′H and seven other F3′5′H proteins revealed an 88.69% amino acid sequence similarity. All F3′5′H proteins contain the conserved flavonoid hydroxylase domain, including an N-terminal proline-rich region. Additionally, conserved features such as the proline-rich region, heme-binding domain, oxygen-binding motif, hydroxylation active site (CR1), EXXR motif, and substrate recognition sites (SRS1-6) were identified. A typical heme-binding motif, FXXGXRXCXG, was found at the C-terminus. Overall, the F3′5′H family members display strong evolutionary conservation (Supplementary Figure S2).

A novel *F3′5′H* gene, designated *RhF3′5′H* (GenBank accession no. PQ523386), was amplified from *Rhododendron × hybridum* using specific primers. The gene’s ORF is 1553 bp, with a GC content of 56%, encoding a 510-amino acid protein (Supplementary Figure S1). ProtParam analysis predicted a molecular weight of ~56.57 kDa, a pI of 8.81, and a stable protein with an instability index of 38.09. It contains 52 acidic and 57 basic residues, indicating a basic nature, and has a hydrophilic character (aliphatic index: 91.27, GRAVY: −0.055). NCBI-CDD analysis confirmed a conserved CYP domain, classifying RhF3′5′H as a cytochrome P450 enzyme. Secondary structure prediction showed 49.02% α-helices and 39.22% random coils, with no β-sheets detected (Supplementary Figure S3). Tertiary modeling identified three conserved motifs: “KLPPGP” (position 48 bp), linking the membrane anchor to the globular enzyme domain; “AGTDTS” (position 334 bp), associated with the oxygen-binding domain; and “PFGAGRRICAG” (position 469 bp), corresponding to the heme-binding domain (Supplementary Figure S4). The constructed pCAMBIA1302-*RhF3′5′H*-GFP vector was introduced into *Agrobacterium tumefaciens*, which was used to infiltrate *Nicotiana benthamiana* epidermal cells. The control GFP construct showed fluorescence throughout the cell, while the RhF3′5′H–GFP fusion protein localized exclusively at the plasma membrane (Figure 5H).

### 2.6. F3′5′H Enhances the Accumulation of Total Anthocyanins

Recombinant and empty vector-transformed E. coli cultures were shaken at 37 °C for 2 h, and when OD_600_ reached ~0.5, IPTG was added at concentrations of 0.1, 0.2, 0.4, 0.8, 1.0, and 2.0 mmol/L to induce protein expression at 16 °C, 28 °C, or 37 °C for 4 h, or overnight at 16 °C. SDS–PAGE (12%) analysis showed that the empty vector carrying the MBP tag produced a ~60 kDa protein, whereas the recombinant plasmid yielded a ~110 kDa fusion protein under the same induction conditions. After MBP tag removal, the target protein appeared at ~56 kDa, matching the predicted size, confirming successful expression of the RhF3′5′H recombinant protein in *E. coli* Rosetta. No target band was observed without IPTG. The target protein band was most intense at 28 °C, indicating that overnight induction at 28 °C with 0.4 mmol/L IPTG is optimal for RhF3′5′H expression (Supplementary Figure S5).

**Figure 6:**
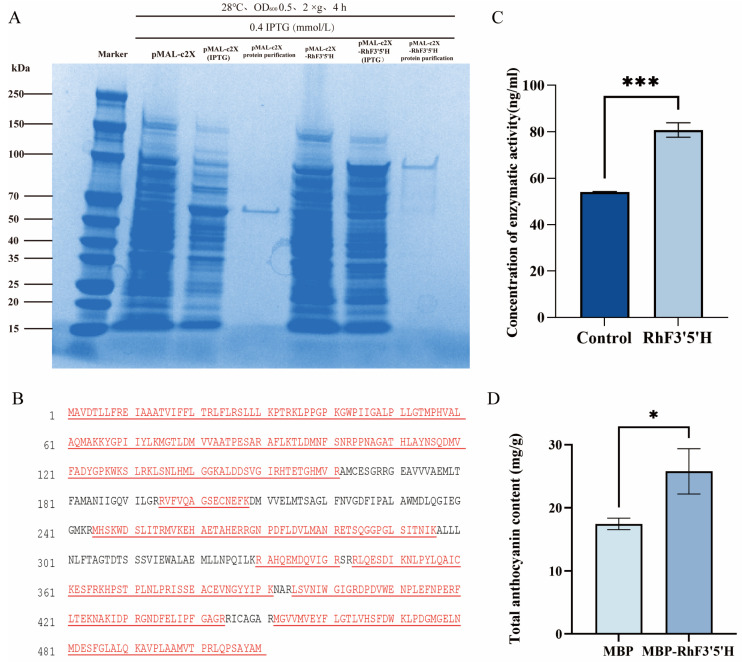
The recombinant RhF3′5′H protein promotes total anthocyanin accumulation. (**A**) SDS–PAGE analysis of MBP–RhF3′5′H expression and purification in *E. coli*. Lane 1, protein marker; Lane 2, pMAL-c2X; Lane 3, IPTG-induced pMAL-c2X; Lane 4, purified protein from pMAL-c2X; Lane 5, pMAL-c2X-RhF3′5′H; Lane 6, IPTG-induced pMAL-c2X-RhF3′5′H; Lane 7, purified MBP–RhF3′5′H fusion protein. (**B**) LC–MS peptide identification of the recombinant protein aligned with the RhF3′5′H amino acid sequence. (**C**) ELISA detection of recombinant RhF3′5′H protein. Data are presented as mean ± SD (n = 3). Significant differences between the two groups were analyzed using Student’s *t*-test. *** *p* < 0.001. (**D**) Total anthocyanin content after treatment with recombinant RhF3′5′H protein. Data are presented as mean ± SD (n = 3). Significant differences between the two groups were analyzed using Student’s *t*-test. * *p* < 0.05.

Both the recombinant and empty vector proteins, which carried an MBP tag and were present in the supernatant, were purified using the PurKine^TM^ Maltose-Binding Protein Tag Protein Purification Kit. The target protein was affinity-purified on PurKine^TM^ Maltose-Binding Protein Tag Dextrin Resin, and eluates were analyzed by 12% SDS-PAGE (Figure 6A). The chromatographic eluates were subjected to LC-MS/MS for data-dependent acquisition, identifying 684 peptides. Compared to the predicted RhF3′5′H sequence, the peptide coverage was 77.65%, confirming the protein as recombinant RhF3′5′H (Figure 6B). Further ELISA analysis revealed that the RhF3′5′H recombinant protein specifically bound to the F3′5′H antibody, with a detected concentration of approximately 80 ng/mL, significantly higher than the control group (Figure 6C).

The study “Complete Biosynthesis of Anthocyanins Using *E. coli*” [18] demonstrated that multiple plant enzymes can be used to reconstruct the biosynthetic pathway in microorganisms, starting from substrates to produce anthocyanins. To preliminarily examine whether the purified RhF3′5′H preparation was associated with flavonoid-related activity, a semi-in vitro petal homogenate assay was performed. The purified recombinant protein was added to ground red *Rhododendron × hybridum* petals and incubated for 2 h, after which the total anthocyanin signal was measured. The RhF3′5′H-treated samples showed a significantly higher anthocyanin signal than the control group (Figure 6D). However, because no exogenous NADPH, cytochrome P450 reductase, or defined flavonoid substrate was supplied, this assay was interpreted cautiously as supportive rather than definitive evidence of catalytic activity.

**Figure 7:**
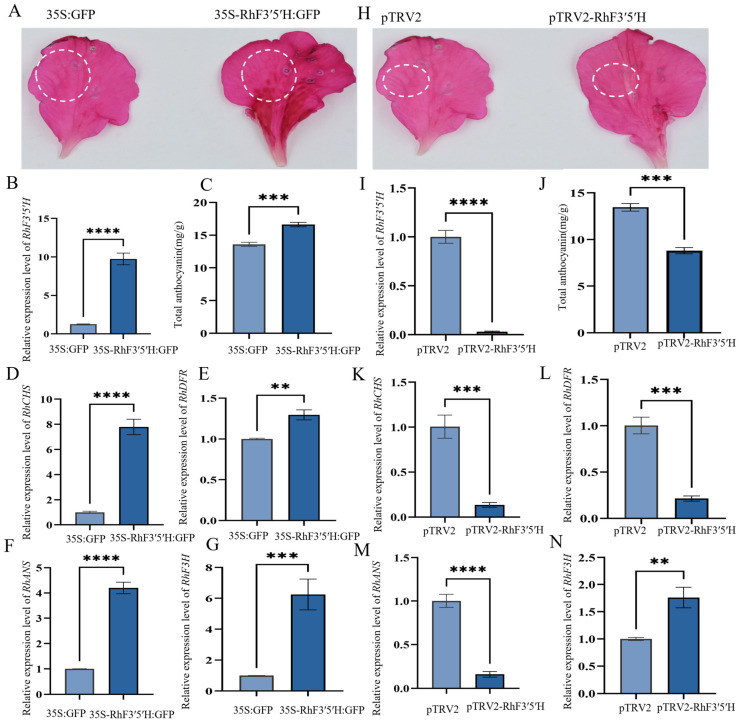
Effects of RhF3′5′H overexpression and silencing on anthocyanin accumulation and the expression of key anthocyanin-biosynthetic genes in *Rhododendron × hybridum* petals. (**A**–**H**) Phenotypic changes in petals after RhF3′5′H overexpression and silencing, respectively. Dashed circles indicate the infiltrated regions. (**B**) Relative expression level of RhF3′5′H in overexpression-treated petals. (**C**) Total anthocyanin content in overexpression-treated petals. (**D**–**G**) Relative expression levels of *CHS*, *F3H*, *DFR*, and *ANS* after RhF3′5′H overexpression. (**I**) Relative expression level of RhF3′5′H in silenced petals. (**J**) Total anthocyanin content in silenced petals. (**K**–**N**) Relative expression levels of *CHS*, *F3H*, *DFR*, and *ANS* after RhF3′5′H silencing. Data are presented as mean ± SD (n = 3). Significant differences between the two groups in each panel were analyzed using Student’s *t*-test. ** *p* < 0.01, *** *p* < 0.001, **** *p* < 0.0001.

Molecular docking analysis indicated that RhF3′5′H binds strongly to naringenin (ΔG = −7.8 kcal/mol) and dihydrokaempferol (ΔG = −8.1 kcal/mol), featuring prominent hydrogen-bonding and hydrophobic interactions (Supplementary Figure S6). Transient overexpression and gene-silencing assays were conducted in petals of red *Rhododendron × hybridum*. qPCR analysis showed that RhF3′5′H transcript abundance increased by ~9.74-fold in overexpression-treated petals compared with the pCAMBIA1302 control (*p* < 0.001; Figure 7B). Consistently, total anthocyanin content was 1.25-fold higher in RhF3′5′H-overexpressing petals than in the control (*p* < 0.0001; Figure 7C). Moreover, the expression of key anthocyanin-biosynthetic genes (*CHS*, *F3H*, *DFR* and *ANS*) was up-regulated in the overexpression samples (Figure 7D–G). In the silencing assay, suppression of RhF3′5′H decreased anthocyanin content by 1.53-fold relative to the pTRV control (*p* < 0.001; Figure 7I,J). Accordingly, transcript levels of CHS, DFR and ANS were reduced, whereas F3H expression increased in silenced petals (Figure 7K–N).

## 3. Discussion

This study systematically identified the *CYP75* gene family in *Rhododendron simsii* and comprehensively described the function of the RhF3′5′H gene in *Rhododendron × hybridum*, revealing the molecular mechanisms of anthocyanin biosynthesis and flower color regulation in *Rhododendron*. These findings provide a useful initial framework for the characterization of the CYP75 gene family in Rhododendron and identify RhF3′5′H as a promising candidate for future studies on anthocyanin regulation and flower-color improvement.

This clustered distribution is a typical feature of gene family expansion and a key driver of functional diversification in plants [19]. Structural analysis confirmed that all RsCYP75 proteins contain the conserved cytochrome P450 domain and the typical heme-binding motif (FXXGXRXCXG), which are characteristic of flavonoid B-ring hydroxylases involved in anthocyanin biosynthesis [20]. The RsCYP75 family is divided into A and B subfamilies, each with distinct motif compositions and cis-regulatory elements, reflecting functional diversification during evolution. The *RsCYP75A* subfamily is enriched with hormone-responsive and stress-related elements, while the *RsCYP75B* subfamily is more associated with growth and development-related elements. This promoter architecture is consistent with the emerging view that transcription factors integrate hormone and stress cues to coordinate secondary metabolism and stress-responsive gene expression in plants [21]. This divergence suggests that *RsCYP75* genes may be involved in multiple physiological processes beyond flower color regulation, such as stress response and hormone signaling, consistent with the functional diversity of the CYP75 family reported in *Orchidaceae* [13] and *Vitaceae* [22]. Compared to the CYP75 family members in herbaceous plants, the RsCYP75 family in Rhododendron shows many structural similarities, but its chromosomal distribution exhibits significant differences. For example, in Petunia, the CYP75 family is distributed across multiple chromosomes without significant gene clustering [10], while in Rhododendron, *RsCYP75* genes form a distinct cluster on chromosome 13. This difference may be linked to the unique evolutionary pressures faced by woody plants, such as long-term environmental adaptation and complex developmental regulation, suggesting that the CYP75 family in azaleas has undergone lineage-specific evolution [23]. The identification of these genes provides a genome-wide framework for the initial characterization of the *CYP75* gene family in *Rhododendron* and lays the foundation for future studies on the specific roles of individual members in flower-color regulation and other aspects of secondary metabolism.

Spatio-temporal expression analysis showed that *RhF3′5′H* (designated as *RhCYP75A2*) exhibits clear tissue specificity in *Rhododendron × hybridum*. In both red and pink Belgian azalea petals, the expression level of *RhF3′5′H* was markedly higher in red petals at the S3 stage, which was consistent with the dynamic pattern of anthocyanin accumulation. This expression pattern is in agreement with reports in other ornamental plants, such as *Brunfelsia acuminata* [17] and *Platycodon grandifloras* [24], supporting the close association between *F3′5′H* expression and anthocyanin biosynthesis. More importantly, the gain- and loss-of-function assays provided direct functional evidence that *RhF3′5′H* participates in anthocyanin regulation in *Rhododendron × hybridum* petals (Figure 7). Transient overexpression of *RhF3′5′H* significantly increased anthocyanin accumulation and up-regulated the expression of several key structural genes in the anthocyanin biosynthetic pathway, whereas silencing of *RhF3′5′H* produced the opposite effect on anthocyanin content and reduced the expression of *CHS*, *DFR*, and *ANS* (Figure 7C–N). Notably, *F3H* transcript abundance increased after RhF3′5′H silencing, suggesting that perturbation of *RhF3′5′H* may trigger compensatory or feedback regulation within the upstream flavonoid pathway. The increased expression of F3H after RhF3′5′H silencing may reflect compensatory plasticity in the upstream flavonoid pathway, which is consistent with recent evidence that F3H family members can exhibit diverse regulatory and stress-related functions in crops [25]. This non-parallel transcriptional response indicates that anthocyanin biosynthesis in azalea petals is regulated not only by the activity of a single structural gene, but also by dynamic interactions among multiple pathway components. In addition, although *RhF3′5′H* overexpression caused a marked increase in transcript abundance, the corresponding increase in total anthocyanin content was relatively moderate, implying that anthocyanin accumulation may still be constrained by substrate availability, metabolic flux, or competition with other branch-pathway enzymes such as F3′H. Although transient overexpression of RhF3′5′H significantly increased total anthocyanin content, the magnitude of the increase was modest (1.25-fold), whereas silencing caused a more pronounced reduction (1.53-fold). This asymmetry suggests that RhF3′5′H is important for maintaining anthocyanin biosynthetic output under native conditions, but that overexpression of this single gene alone is insufficient to produce a large gain in pigment accumulation or an obvious shift in flower color. In this context, the biological significance of the overexpression result should be interpreted mainly at the pathway level rather than as a direct predictor of visible color change. The limited gain in anthocyanin accumulation may reflect constraints imposed by substrate availability, competition with F3′H for shared intermediates, downstream enzymatic capacity, or cellular factors affecting pigment stabilization and storage. Moreover, the absence of detectable delphinidin-derived blue pigmentation is not inconsistent with the positive role of RhF3′5′H in the pathway, because blue flower formation depends not only on F3′5′H activity, but also on the relative cyanidin/delphinidin balance, vacuolar pH, co-pigmentation, metal ion complexation, and petal epidermal cell structure. Therefore, the contribution of *RhF3′5′H* to flower coloration should be understood within the broader context of pathway coordination rather than as an isolated single-gene effect. Compared with other species, *RhF3′5′H* also exhibits a distinct expression pattern. For example, F3′5′H genes in red grapes and the medicinal herb *Epimedium* are expressed in multiple tissues [26,27], whereas *RhF3′5′H* is predominantly expressed in petals, suggesting a more specialized role in flower color formation. This tissue-specific expression may be associated with cis-regulatory elements in the *RhF3′5′H* promoter, such as abundant light-responsive and MeJA-responsive elements, which are known to regulate anthocyanin biosynthesis genes in response to environmental and hormonal signals [21,28]. The enrichment of MeJA-responsive elements in RsCYP75A promoters also suggests potential regulation by jasmonate-related signaling, which is increasingly recognized as an important coordinator of defense-associated secondary metabolism [29]. As a member of the *CYP75A* subfamily, RhF3′5′H contains all conserved functional domains, including the proline-rich region, heme-binding domain, EXXR motif, and substrate recognition sites (SRS), which are crucial for the catalysis of B-ring 3′,5′-hydroxylation in flavonoids [22,30].

The construction and optimization of the prokaryotic expression system, together with subcellular localization analysis, provided additional support for the functional characterization of RhF3′5′H. Subcellular localization analysis showed that RhF3′5′H was predominantly localized to the plasma membrane, which is consistent with the membrane-associated nature of cytochrome P450 proteins [31]. Subcellular localization analysis showed that RhF3′5′H was predominantly localized to the plasma membrane, which is consistent with the membrane-associated nature of cytochrome P450 proteins. This localization pattern is also supported by the structural prediction of RhF3′5′H. In particular, the predicted motif linking the membrane-anchor region to the globular enzyme domain suggests a membrane-associated topology in which the catalytic domain is appropriately positioned relative to the membrane. Although the exact topology was not experimentally resolved in this study, the agreement between the in-silico prediction and the RhF3′5′H–GFP localization result strengthens the interpretation that membrane association is an intrinsic structural feature of RhF3′5′H. The pMAL-c2X vector was used to generate an MBP-tagged fusion construct, and the MBP tag likely contributed to improved protein solubility. The pMAL-c2X vector was used to generate an MBP-tagged fusion construct, and the MBP tag likely contributed to improved protein solubility [32,33]. SDS–PAGE analysis showed that the target protein was mainly detected in the soluble fraction. The recombinant plasmid was expressed in *Escherichia coli* Rosetta, a host commonly used for heterologous protein expression in bacterial systems. Under the optimized induction conditions, namely 0.4 mmol/L IPTG at 28 °C, the highest proportion of soluble recombinant protein was obtained. The recombinant plasmid was expressed in *Escherichia coli* Rosetta, a host commonly used to improve heterologous expression of eukaryotic genes in bacterial systems. Under the optimized induction conditions, namely 0.4 mmol/L IPTG at 28 °C, the highest proportion of soluble recombinant protein was obtained. Subsequent SDS–PAGE, LC–MS/MS, and ELISA analyses confirmed the successful purification and identity of the recombinant RhF3′5′H protein. In addition, molecular docking suggested that RhF3′5′H can interact with naringenin and dihydrokaempferol through hydrogen-bonding and hydrophobic interactions. Together, these results indicate that the recombinant RhF3′5′H protein was successfully expressed in a soluble form and retained structural features consistent with its proposed role as a flavonoid B-ring hydroxylase, thereby providing experimental support for its functional involvement in anthocyanin biosynthesis. It should be noted that the semi-in vitro petal homogenate assay used in this study was not a fully reconstituted cytochrome P450 enzymatic system, because no exogenous NADPH, cytochrome P450 reductase, or defined flavonoid substrate was added. Therefore, this experiment should not be interpreted as direct biochemical proof that RhF3′5′H catalyzes flavonoid hydroxylation under the assay conditions used here. Rather, it provides preliminary supportive evidence that the purified RhF3′5′H preparation is associated with an increased anthocyanin signal in petal homogenates. In the present study, the strongest functional support for RhF3′5′H comes from the in-planta transient overexpression and gene-silencing analyses assays, together with its conserved F3′5′H motifs and substrate-binding predictions from molecular docking. Future studies should reconstitute RhF3′5′H activity in a complete redox-supported system and directly identify hydroxylated products by LC–MS.

A significant finding of this study is that, despite the high expression of *RhF3′5′H* in the red *Rhododendron × hybridum* petals, no significant blue pigment (such as delphinidin) was detected in the petals. This result seems to contradict the well-known role of F3′5′H in promoting blue or purple flower color in other species [17,34]. This paradox can be explained by the metabolic competition between F3′5′H and F3′H in the anthocyanin biosynthesis pathway. F3′H catalyzes the conversion of DHK to DHQ, a precursor of cyanidin (red), which competes with F3′5′H for the common substrate DHK, leading to changes in the accumulation of delphinidin and consequently affecting flower color [35]. The formation of flavonoid substrate channels or protein–protein metabolons, which have been documented in *Arabidopsis* [31] and *Petunia* [17], could play a role in channeling substrates more efficiently through the anthocyanin biosynthetic pathway. These complexes facilitate the direct transfer of substrates between enzymes, thereby enhancing catalytic efficiency and mitigating substrate limitations. By optimizing substrate utilization, these metabolons could help balance the competition between *F3′5′H* and *F3′H*, ensuring that substrates are more effectively directed toward the desired anthocyanin products. In studies of *Rhododendron × hybridum* petals at the full bloom stage, cyanidin content was found to be approximately 3403.40 ng/g in fresh petal weight, while delphinidin content was 235.04 ng/g [36], which is consistent with the relative expression analysis of the aforementioned genes, mutually corroborating the experimental conclusions. In contrast, a multi-gene co-regulation strategy is more feasible for creating blue flowers. However, although various methods have been used to enhance delphinidin accumulation, flower color does not necessarily turn blue, as the final petal color is influenced by many factors, including vacuolar pH, metal ions, co-pigments, and cellular structure [37]. Therefore, the mechanisms underlying the formation of blue petals are complex, and further studies are needed to explore the molecular regulation of anthocyanin biosynthesis [38].

## 4. Materials and Methods

### 4.1. Experimental Materials

The plant material used in this study included the petals of *Rhododendron × hybridum* (red cultivar and pink cultivar) at the full-bloom stage (S3), as well as roots, stems, leaves from red cultivar, Fresh and clean petals of the whole flower in three developmental periods including the bud stage (S1), the nascent stage (S2), the full-blooming stage (S3) were respectively collected from *Rhododendron × hybridum*, which grew in Ningbo Wanjing Azalea Garden, Ningbo, China. *Nicotiana benthamiana* seedlings at 4–6 weeks of age were used in the experiments.

### 4.2. Rationale for Combining Genome Analysis in R. simsii with Functional Assays in R. × hybridum

*Rhododendron simsii* and *Rhododendron × hybridum* are not the same taxon. *R. simsii* was used as the reference species for genome-wide identification of CYP75 family members because a high-quality chromosome-level genome is available for this species, and *R. simsii* has been recognized as a major ancestral species of cultivated *Rhododendron*. In contrast, *R. × hybridum* was used for expression and functional analyses because the cultivars used in this study are horticulturally relevant materials and are suitable for petal-based transient assays. The candidate gene RhF3′5′H was cloned directly from *R. × hybridum* cDNA as the putative homolog of *RsCYP75A2* identified from the *R. simsii* genome, based on sequence similarity, conserved motifs, and phylogenetic clustering within the CYP75A/F3′5′H clade.

### 4.3. Identification of CYP75 Gene Family Members

In this study, CYP family A and B subfamily sequences from 50 species were downloaded from NCBI: https://www.ncbi.nlm.nih.gov (accessed on 17 July 2025). BLAST + 2.17.0 searches were then performed against the *Rhododendron simsii* genome reported by Yang et al. [39]. The final assembly is available under accession WJXA00000000. Conserved domains of the predicted CYP75 proteins were retrieved from the InterPro database: https://www.ebi.ac.uk/interpro/ (accessed on 29 January 2026), and profile HMMs were subsequently constructed for genome-wide identification. And perform a genome-wide search against the *Rhododendron* genome using HMMER 3.0, with a cutoff threshold of E-value ≤ 1 × 10^−5^ to identify candidate *CYP75* genes.

### 4.4. Gene Structure and Evolutionary Analysis of the CYP75 Gene Family Members

Conserved motifs in *RsCYP75* protein sequences were identified using MEME (https://meme-suite.org/meme/tools/meme, accessed on 17 July 2025), with the parameter set to predict 10 motifs (other parameters were default).

### 4.5. Chromosomal Localization of the CYP75 Gene Family

The positional annotation data for the *RsCYP75* genes were extracted from the GFF (General Feature Format) file of the *R. simsii* genome annotation to analyze their chromosomal distribution patterns. These genes were subsequently renamed based on their chromosomal positions.

### 4.6. Phylogenetic Analysis of the CYP75 Gene Family Members

The CYP75 protein sequences from the *Rhododendron* genome and 50 other species were aligned using MEGA 11 software for multiple sequence alignment [40]. A phylogenetic tree was constructed by the neighbor-joining (NJ) method, with 1000 bootstrap replicates to assess the tree reliability. The resulting tree file was imported into the online tool Evolview (https://evolgenius.info, accessed on 17 July 2025) for beautification and editing.

### 4.7. Prediction of Physicochemical Properties and Protein Structure of CYP75 Proteins

For cis-acting regulatory elements (CAREs) prediction, a 2000 bp Nucleotide Sequence upstream of the start codon (ATG) of each *RsCYP75* gene was extracted, and the sequences were submitted to the PlantCARE database (https://bioinformatics.psb.ugent.be/webtools/plantcare/html/, accessed on 17 July 2025) for CAREs prediction. Subsequently, the predicted cis-acting regulatory elements were visualized using TBtools1.6 software [41].

### 4.8. qRT-PCR Analysis

Total RNA was extracted from the flowers at S1–S3 stages of *Rhododendron × hybridum* (red cultivar and pink cultivar) and roots, stems, and leaves, using a plant RNA extraction kit (Vazyme, Nanjing, China). Approximately 0.1 g of each tissue sample was ground, and RNA extraction was performed according to the manufacturer’s instructions. The RNA concentration was measured, and 10 μL of RNA at a concentration of 100 ng/μL was reverse-transcribed into cDNA using PrimeScript^TM^ RT Master Mix (Takara, Tokyo, Japan). The resulting cDNA was used as the template for subsequent PCR amplification. Following the experimental protocol established by Jia et al. [36], quantitative PCR was conducted using SYBR qPCR Master Mix (Vazyme, Beijing, China) by quantitative real-time PCR (qRT-PCR) using BioRad CFX96 Real-Time PCR System (Biorad, Berkeley, CA, USA), with each treatment comprising three technical replicates. The Ct value of the *CYP75* gene was assessed using the 2^−ΔΔCT^ method. All experimental data were technologically repeated 3 times. Results were presented as mean ± standard deviation (S.D.). All primers used in this study are listed in Supplementary Table S1.

### 4.9. Physicochemical Properties and Protein Structure Analysis of CYP75

The CYP75 gene and its deduced amino acid sequence were analyzed using AlignX software. The theoretical molecular weight and isoelectric point (pI) were calculated via the ProtParam tool (http://web.expasy.org/protparam/, accessed on 17 July 2025). The secondary structure of the protein was predicted with SOPMA (https://npsa.lyon.inserm.fr/cgi-bin/npsa_automat.pl?page=/NPSA/npsa_sopma.html, accessed on 17 July 2025), while the tertiary structure was predicted by AlphaFold2 (https://alphafold.com/, accessed on 17 July 2025).

### 4.10. Plasmid Construction

The full length of *RhF3′5′H* was cloned from *Rhododendron × hybridum*’s cDNA. The *RhF3′5′H* was constructed using the PCR. The full-length *RhF3′5′H* was inserted into the *NcoI*-digested plasmid pCMBIA1302-GFP vector, resulting in pCAMBIA1302- *RhF3′5′H-GFP*. pMAL-c2X- RhF3′5′H was constructed by inserting the *RhF3′5′H* CDS into pMAL-c2X via One Step Cloning. Virus-induced gene silencing (VIGS) was carried out using a tobacco rattle virus (TRV)-based system. A 300-bp gene-specific fragment of *RhF3′5′H* was amplified by PCR and subsequently cloned into the pTRV2 vector to generate *pTRV2-RhF3′5′H*. All primers used in this study are listed in Supplementary Table S1.

### 4.11. F3′5′H Multiple Sequence Alignment

The F3′5′H amino acid sequences from various species were obtained from the GenBank database on the NCBI platform. Subsequently, Jalview was utilized to perform multiple sequence alignments of the amino acid sequences belonging to the CYP75 family [42]. Visualization of the results was conducted using DNAMAN (version 8.0, Lynnon Biosoft), a sequence analysis software package for molecular biology applications.

### 4.12. Sub-Cellular Localization

Agrobacterium strain GV3101 containing expression plasmids was centrifuged for 60 s at 8000 rpm, resuspended in buffer (10 mM MES, pH 5.6, 10 mM MgCl_2_, 200 mM acetosyringone), and then diluted to an OD_600_ of 0.8. GV3101 strain containing pCAMBIA1302-*RhF3′5′H-GFP* was resuspended and adjusted to an OD_600_ with infiltration medium before leaf infiltration. Next, it was infiltrated into *N. benthamiana* leaves then cultured at 25 °C for 72 h. The expression of fluorescent proteins was examined at 48 h post agroinfiltration under a Leica TCS SP8 confocal laser scanning microscope (Leica Microsystems, Heidelberg, Germany).

### 4.13. Agrobacterium-Mediated Transient Expression and VIGS Assay

Agrobacterium cultures (1:50 dilution) were grown in antibiotic-supplemented LB medium at 28 °C with shaking at 200 rpm for 12–16 h, harvested by centrifugation (5000 rpm), and resuspended in infiltration buffer (10 mM MgCl_2_, 10 mM MES, and 0.1 mM acetosyringone; pH 5.8) to an OD_600_ of 0.8. For transient overexpression, petals of red *Rhododendron × hybridum* were injected with Agrobacterium carrying *pCAMBIA1302-RhF3′5′H-GFP* or the corresponding empty-vector control pCAMBIA1302-GFP. For VIGS, Agrobacterium carrying *pTRV2-RhF3′5′H* (or pTRV2 as a control) was mixed with pTRV1 at a 1:1 ratio and injected into petals. After infiltration, treated petals were enclosed in opaque bags and kept in darkness for 3 days; the bags were then removed and samples were collected.

### 4.14. Protein Induction and Purification

Protein induction was performed using IPTG as described previously [43]. Recombinant and empty plasmids were transformed into *E. coli* Rosetta cells, and positive clones were selected via resistance screening. A positive colony was cultured in 5 mL LB medium with 50 mg/L ampicillin at 37 °C for 2 h. When OD_600_ reached 0.5, IPTG was added to 0.5 mmol/L, and induction was carried out at 28 °C for 4 h. To optimize soluble protein expression, IPTG concentrations (0.1–2.0 mmol/L) and temperatures (28 °C, 37 °C, 16 °C) were tested. After induction, 500 μL of culture was centrifuged, and the pellet was resuspended in 200 μL of 0.1% PBS. Protein samples were mixed with loading buffer, heated at 100 °C for 10 min, and analyzed by 12% SDS-PAGE.

Two 250 mL sterile conical flasks were prepared, and bacterial solutions with recombinant pMAL-c2X-RhF3′5′H and empty plasmid pMAL-c2X were added to LB medium with 50 mg/L ampicillin at a 1:100 dilution. The cultures were incubated at 37 °C with shaking at 200 rpm for 2 h. When OD_600_ reached 0.5, IPTG was added to 0.5 mmol/L, and induction was carried out at 28 °C for 4 h. After centrifugation at 8000 rpm for 5 min, the pellet was resuspended in 3 mL of 0.1% PBS. Ultrasonic disruption was applied for 15 min, followed by centrifugation at 10,000 rpm for 10 min. The supernatant was collected and purified using the PurKine^TM^ Maltose Binding Protein Purification Kit (Dextrin; KTP2020, Abbkine, Wuhan, China), which is based on dextrin affinity purification of MBP-tagged fusion proteins, according to the manufacturer’s instructions. The purified protein was mixed with protein loading buffer, heated at 100 °C for 10 min, and analyzed by 12% SDS-PAGE.

### 4.15. Identification of the Recombinant Protein

Target protein identification was performed by Liquid Chromatography-Tandem Mass Spectrometry (LC-MS/MS). The target protein bands obtained from SDS-PAGE were processed for LC-MS/MS analysis using the following steps: (1) Band decolorization: Gel bands were cut, treated with a mixed solution of acetonitrile and ammonium bicarbonate, and dried with 100% acetonitrile. (2) Reductive alkylation: Bands were treated with dithiothreitol (DTT) at 56 °C, followed by iodoacetamide (IAM) treatment in the dark, then air-dried. (3) In-gel digestion: Bands were digested with trypsin at 37 °C for 16 h. (4) Peptide extraction: Peptides were extracted with trifluoroacetic acid (TFA), acetonitrile, and water, then vacuum-dried. (5) Desalting: Samples were desalted using a commercial Peptide Desalting C18 StageTip (Cell Signaling Technology, Danvers, MA, USA) for peptide cleanup prior to LC–MS/MS analysis and were then vacuum-dried. LC-MS/MS analysis was performed with a Reprosil-Pur C18-AQ (Dr. Maisch HPLC GmbH, Ammerbuch-Entringen, Germany), which is an octadecyl-bonded silica column with aqueous compatibility for peptide separation.

Protein identification via ELISA was performed as described by Li et al. [44] with minor modifications. The purified recombinant protein was used as the sample, and its concentration was determined using the Plant Flavonoid 3′,5′-hydroxylase ELISA Kit (Ningbo Kangsheng Biotechnology Co., Ltd., Ningbo, China). After equilibration of the kit to room temperature for 20 min, the required microplate strips were removed from the sealed pouch, while the remaining strips were resealed and stored at 4 °C. Standard and sample wells were set up, with 50 μL of standard solutions added to the standard wells. Then, 10 μL of the sample and 40 μL of diluent were added to the sample wells, while the blank wells were left untreated. After adding 50 μL of stop solution to each well, OD values were measured at 450 nm within 15 min using a SPECTRAMAX190 Microplate Reader (Molecular Devices, San Jose, CA, USA).

### 4.16. Molecular Docking Analysis

The 3D structures of the naringenin (CAS:480-41-1) and dihydrokaempferol (CAS:480-20-6) ligands were downloaded from the PubChem database: https://pubchem.ncbi.nlm.nih.gov/ (accessed on 10 May 2025). For receptor and ligand preparation and molecular docking analysis, AutoDock 4.2 and AutoDock Vina were employed. AutoDock 4.2 was used for docking setup and grid-based calculations, whereas AutoDock Vina was used to predict ligand-binding conformations and binding affinities [45]. The docking was performed using a cubic grid box with dimensions of 40 Å × 40 Å × 40 Å, centered at coordinates (center x = −0.463, center y = −0.704, center z = −0.077). After docking, the binding modes and intermolecular interactions between RhF3′5′H and the ligands naringenin and dihydrokaempferol were further analyzed and visualized using PyMOL 2.5, PLIP, and LigPlot+ v.2.3 software [46].

### 4.17. Measurement of Petal Color Phenotype

Petal color at full bloom was assessed using the Royal Horticultural Society Colour Chart (RHSCC) in *Rhododendron × hybridum* (red and pink cultivars). In addition, petal color of the red cultivar was compared between the transient overexpression and VIGS treatments and their corresponding controls following the method described by Xu et al. [47]. Color parameters were measured using a high-quality computer colorimeter (Focus on Color 3nh, 3nh Intelligent Technology Co., Ltd., Guangzhou, China.) with the CIELAB system. The color coordinates were as follows: L* for lightness, ranging from black (0) to white (100); a* for the red (positive) to green (negative) axis; and b* for the yellow (positive) to blue (negative) axis for each line, three fully open flowers were randomly selected for investigating color differences, as shown in Supplementary Table S3. The scoring was repeated three times.

### 4.18. Quantification of Total Anthocyanin Content

Total anthocyanins were extracted from petal samples following Wu et al. [35]. The petal samples were frozen in liquid nitrogen and then ground into a fine powder. Approximately 0.2 g of the powdered petal sample was used for further analysis. The sample was mixed with 1 mL of 1% hydrochloric acid-methanol (1:99) solution and shaken at room temperature for 18 h. The mixture was then centrifuged at 12,000 rpm for 5 min. From the supernatant, 400 μL was combined with 600 μL of the hydrochloric acid-methanol solution. The absorbance was measured at 530 nm and 657 nm using a SPECTRAMAX190 Microplate Reader (Molecular Devices, USA). The total anthocyanin content was calculated using the formula: (A530 − 0.25 × A657)/m, where m is the sample mass in grams.

To investigate the effect of the recombinant protein on the total anthocyanin content, following the method described above, the recombinant protein was added to 0.1 g of ground red *Rhododendron × hybridum* flowers and incubated in a water bath at 25 °C for 2 h. For the semi-in vitro assay, no exogenous flavonoid substrate, NADPH, or cytochrome P450 reductase was added; therefore, the assay reflected the interaction of the purified recombinant protein preparation with endogenous components present in the petal homogenate. The absorbance was then measured at 530 nm and 700 nm using a SPECTRAMAX190 Microplate Reader (Molecular Devices, USA).

## 5. Conclusions

In this study, a comprehensive analysis of the *CYP75* gene family in *Rhododendron simsii* was conducted, with a particular focus on the functional characterization of *RhF3′5′H* in *Rhododendron × hybridum*. Seven *RsCYP75* genes were identified, including a prominent gene cluster on chromosome 13, and were classified into two conserved subfamilies (*CYP75A* and *CYP75B*). *RhF3′5′H*, which showed the highest expression in red petals, was validated as a functional F3′5′H-type enzyme involved in anthocyanin biosynthesis. Transient overexpression of *RhF3′5′H* in red petals markedly elevated its transcript abundance and increased total anthocyanin content, accompanied by the up-regulation of key structural genes in the anthocyanin pathway (*CHS*, *F3H*, *DFR* and *ANS*). Conversely, *RhF3′5′H* silencing reduced anthocyanin accumulation and reprogrammed pathway gene expression (*CHS*, *DFR* and *ANS* decreased, whereas *F3H* increased), providing complementary loss-of-function evidence and suggesting feedback regulation within the upstream flavonoid network. In addition, RhF3′5′H was successfully expressed in *E. coli* as a soluble recombinant protein that retained catalytic potential for flavonoid B-ring hydroxylation, further supporting its enzymatic functionality. Collectively, these results refine the *CYP75* gene catalog in *Rhododendron* and provide candidate targets and molecular resources for future studies and ornamental breeding aimed at improving flower color. These findings provide useful molecular resources for future ornamental trait improvement and may support rational molecular breeding strategies for flower-color modification in Rhododendron, in line with emerging SMART crop concepts for precision trait design [48].

## Data Availability

No new data were created or analyzed in this study. Data sharing is not applicable to this article.

## Supplementary Materials

The following supporting information can be downloaded at: https://www.mdpi.com/article/10.3390/plants15101472/s1, Figure S1: Multiple sequence alignment and characteristic fragments of F3’5’H proteins; Figure S2: Nucleotide sequence of RhF3’5’H and its encoded amino acid sequence; Figure S3: Secondary structure prediction; Figure S4: Tertiary structure prediction; Figure S5: 12% SDS-PAGE analysis of RhF3’5’H recombinant protein. Figure S6: Molecular Docking of naringenin and dihydrokaempferol with RhF3′5′H and Overexpression of the Gene with Anthocyanin Quantification. Table S1: The sequences of primers used in this study; Table S2: Screening results of CYP75 gene family members in *Rhododendron simsii* genome; Table S3: Petal color classification and CIELAB color parameters of *Rhododendron × hybridum* under different experimental treatments.
