# Supplementary material for: Genome-Wide Identification of CYP75 Gene Family in Rhododendron simsii and Functional Analysis of Its Role in Promoting Anthocyanin Biosynthesis
Source: Plants (Basel). 2026 May 12;15(10):1472. doi: 10.3390/plants15101472 (PMC13210423; doi:10.3390/plants15101472)

Supplementary Figure S1

Multiple sequence alignment and characteristic fragments of F3'5'H proteins.

Multiple sequence alignment of RhF3′5′H with representative F3′5′H proteins from other plant species. Conserved functional regions, including the N-terminal proline-rich region, substrate recognition sites (SRS1–SRS6), CR1 region, EXXR motif, and the heme-binding domain, are indicated in the alignment. The high level of sequence conservation among these proteins supports the structural conservation of F3′5′H family members.

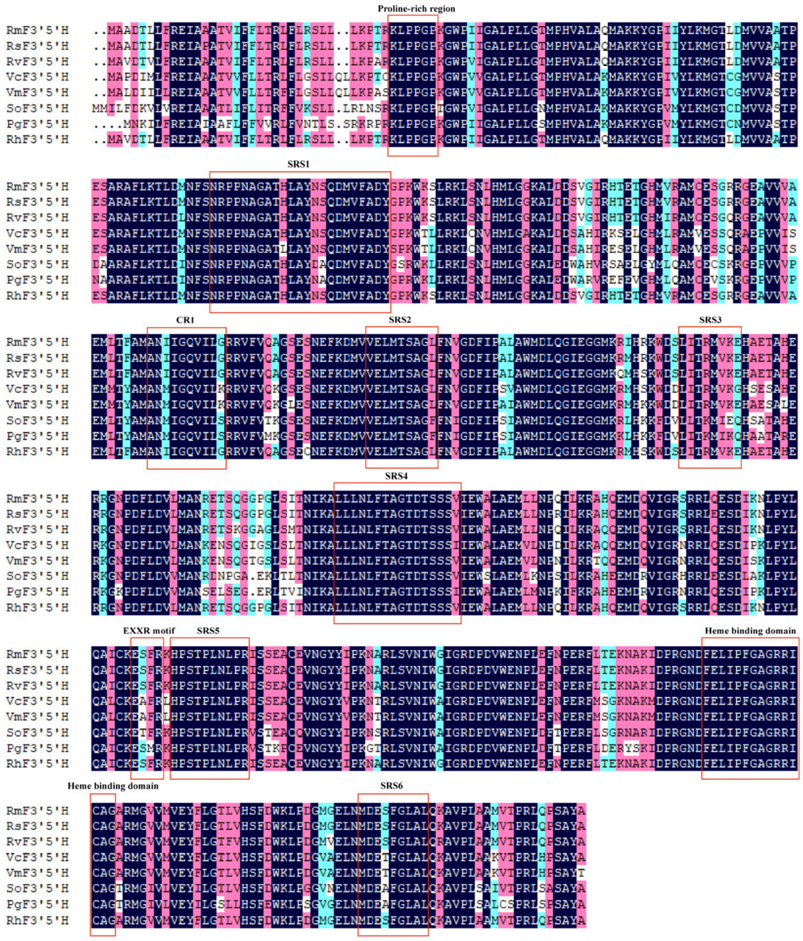

Supplementary Figure S2

Nucleotide sequence of *RhF3'5'H* and its encoded amino acid sequence.

The full-length coding sequence of RhF3′5′H and the corresponding deduced amino acid sequence are shown. The start codon and stop codon delimit the open reading frame, and the translated amino acid residues are presented below the nucleotide sequence.

```
+1    M  A  V  D  T  L  L  F  R  E  I  A  A  A  T  V  I  F  F  L  T  R  L  F  L  R  S  L  L  L  K  P  T  R
      ATGGCCGTAG ACACTCTGTT GTTTAGAGAG ATTGCAGCAG CAACTGTAAT CTTTTTCCTC ACTAGGCTGT TCCTCCGTTC CCTCCTCCTC AAACCCACCC GT
+1    K  G  W  P  I  I  G  A  L  P  L  L  G  T  M  P  H  V  A  L  A  Q  M  A  K  K  Y  G  P  I  I  Y  L  K
121   AAAGGGTGGC CGATCATCGG CGCCCTCCCC CTTCTTGGAA CCATGCCCCA TGTTGCCCTA GCCCAAATGG CCAAGAAATA TGGACCCATC ATCTACCTAA AA
+1    V  V  A  A  T  P  E  S  A  R  A  F  L  K  T  L  D  M  N  F  S  N  R  P  P  N  A  G  A  T  H  L  A  Y
241   GTGGTGGCCG CGACGCCCGA GTCGGCCCGA GCCTTCCTGA AAACCCTGGA CATGAACTTC TCAAACCGGC CACCTAATGC CGGCGCGACC CACTTGGCAT AC
+1    F  A  D  Y  G  P  K  W  K  S  L  R  K  L  S  N  L  H  M  L  G  G  K  A  L  D  D  S  V  G  I  R  H  T
361   TTCGCCGACT ACGGCCCGAA GTGGAAGTCG TTGCGCAAGT TGAGCAACCT GCACATGCTG GGCGGGAAGG CGCTCGACGA CTCGGTTGGC ATCCGGCATA CG
+1    R  A  M  C  E  S  G  R  R  G  E  A  V  V  V  A  E  M  L  T  F  A  M  A  N  I  I  G  Q  V  I  L  G  R
481   CGGGCCATGT GCGAGTCGGG CCGGAGGGGC GAGGCGGTGG TGGTGGCGGA GATGTTGACG TTCGCCATGG CGAACATCAT CGGCCAGGTG ATACTCGGGC GG
+1    G  S  E  C  N  E  F  K  D  M  V  V  E  L  M  T  S  A  G  L  F  N  V  G  D  F  I  P  A  L  A  W  M  D
601   GGTTCGGAGT GCAACGAGTT CAAGGACATG GTGGTGGAGC TGATGACCTC GGCCGGTCTG TTCAACGTGG GCGACTTTAT ACCGGCGCTG GCGTGGATGG AC
+1    G  M  K  R  M  H  S  K  W  D  S  L  I  T  R  M  V  K  E  H  A  E  T  A  H  E  R  R  G  N  P  D  F  L
721   GGGATGAAAC GGATGCATAG CAAGTGGGAC AGTTTGATAA CGAGGATGGT GAAGGAGCAC GCCGAGACGG CTCATGAGCG TCGAGGGAAC CCTGATTTTC TT
+1    R  E  T  S  Q  G  G  P  G  L  S  I  T  N  I  K  A  L  L  L  N  L  F  T  A  G  T  D  T  S  S  S  V  I
841   AGAGAGACTT CTCAAGGCGG CCCCGGGCTT AGCATCACCA ACATTAAAGC ACTCCTTTTG AATCTATTTA CTGCCGGTAC CGATACCTCT TCCAGCGTAA TC
+1    M  L  L  N  P  Q  I  L  K  R  A  H  Q  E  M  D  Q  V  I  G  R  S  R  R  L  Q  E  S  D  I  K  N  L  P
961   ATGCTGCTGA ATCCACAAAT CCTAAAACGG GCACACCAAG AGATGGATCA AGTCATTGGA AGAAGCAGGA GATTACAGGA GTCTGACATT AAAAACCTGC CT
+1    K  E  S  F  R  K  H  P  S  T  P  L  N  L  P  R  I  S  S  E  A  C  E  V  N  G  Y  Y  I  P  K  N  A  R
1081  AAAGAAAGCT TCCGAAAGCA CCCTTCCACC CCCCTCAACC TCCCCCGAAT CTCATCCGAA GCATGCGAGG TGAACGGTTA CTACATACCC AAGAACGCGC GA
+1    G  I  G  R  D  P  D  V  W  E  N  P  L  E  F  N  P  E  R  F  L  T  E  K  N  A  K  I  D  P  R  G  N  D
1201  GGCATCGGGA GGGACCCCGA CGTCTGGGAG AACCCTCTGG AGTTCAACCC GGAGAGGTTT CTGACCGAGA AAAACGCCAA GATCGATCCG CGGGGGAACG AT
+1    G  A  G  R  R  I  C  A  G  A  R  M  G  V  V  M  V  E  Y  F  L  G  T  L  V  H  S  F  D  W  K  L  P  D
1321  GGCGCGGGGC GGAGGATATG CGCGGGGGCT AGGATGGGAG TTGTGATGGT TGAGTACTTC TTGGGCACGT TGGTTCACTC ATTTGACTGG AAATTGCCTG AT
+1    M  D  E  S  F  G  L  A  L  Q  K  A  V  P  L  A  A  M  V  T  P  R  L  Q  P  S  A  Y  A  M  *
1441  ATGGATGAGT CTTTCGGTCT TGCTTTGCAA AAGGCTGTGC CTCTCGCGGC TATGGTTACC CCGCGGCTGC AACCAAGTGC TTATGCTATG TAG
```

Supplementary Figure S3

Secondary structure prediction.

Predicted secondary structure organization of the RhF3′5′H protein. The light blue regions indicate the overall length of the protein sequence, whereas the red region highlights the conserved cytochrome P450 family domain. The predicted protein length is 510 amino acids.

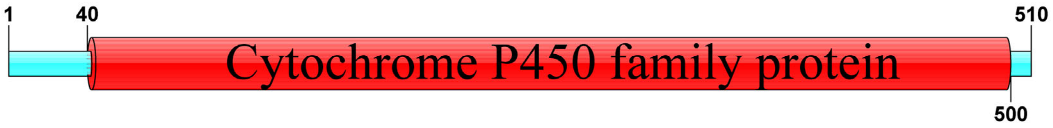

Supplementary Figure S4

Tertiary structure prediction.

Three-dimensional structural model of RhF3′5′H. The conserved proline-rich motif “KLPPGP” is highlighted in red, the EXXR motif and substrate recognition-related motif “AGTDTS” are highlighted in purple, and the heme-binding domain “PTGAGRRICAG” is highlighted in orange. These conserved regions are characteristic features of cytochrome P450 enzymes and support the functional identity of RhF3′5′H as an F3′5′H-type protein. Red indicates the conserved proline-rich motif "KLPPGP," purple denotes the EXXR motif and substrate recognition site "AGTDTS," and orange highlights the heme-binding domain "PTGAGRRICAG".

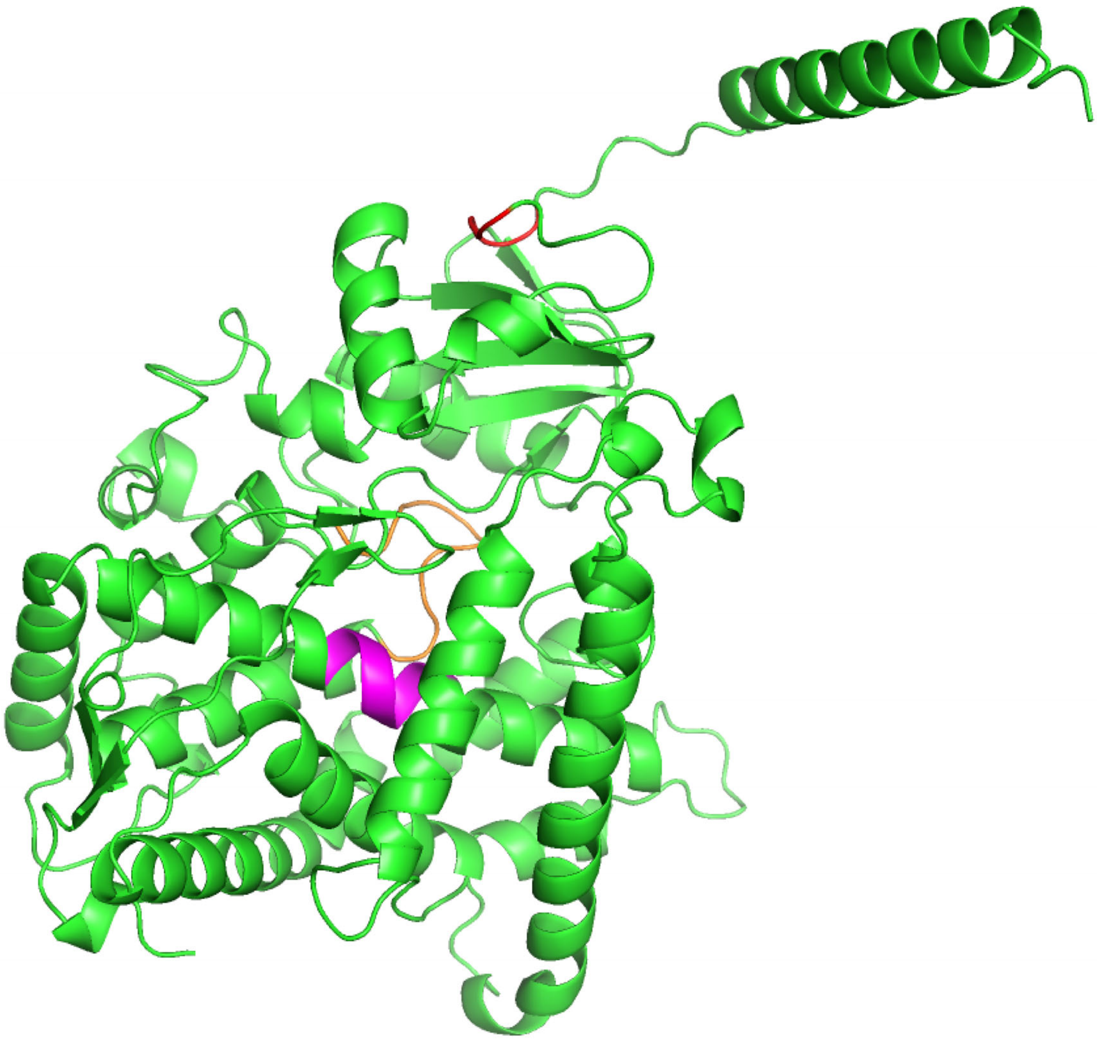

Supplementary Figure S5

12% SDS-PAGE analysis of RhF3'5'H recombinant protein.

(A) 12% SDS–PAGE analysis of recombinant RhF3′5′H protein expression under different induction temperatures. Protein expression was induced in *Escherichia coli* at $OD_{600} \approx 0.5$ with 0.4 mM IPTG, and samples were analyzed after induction under different temperature conditions.(B) 12% SDS–PAGE analysis of recombinant RhF3′5′H protein expression under different IPTG concentrations. Protein expression was induced at 28°C with different IPTG concentrations, and the resulting protein bands were compared to evaluate the optimal induction condition for RhF3′5′H expression.

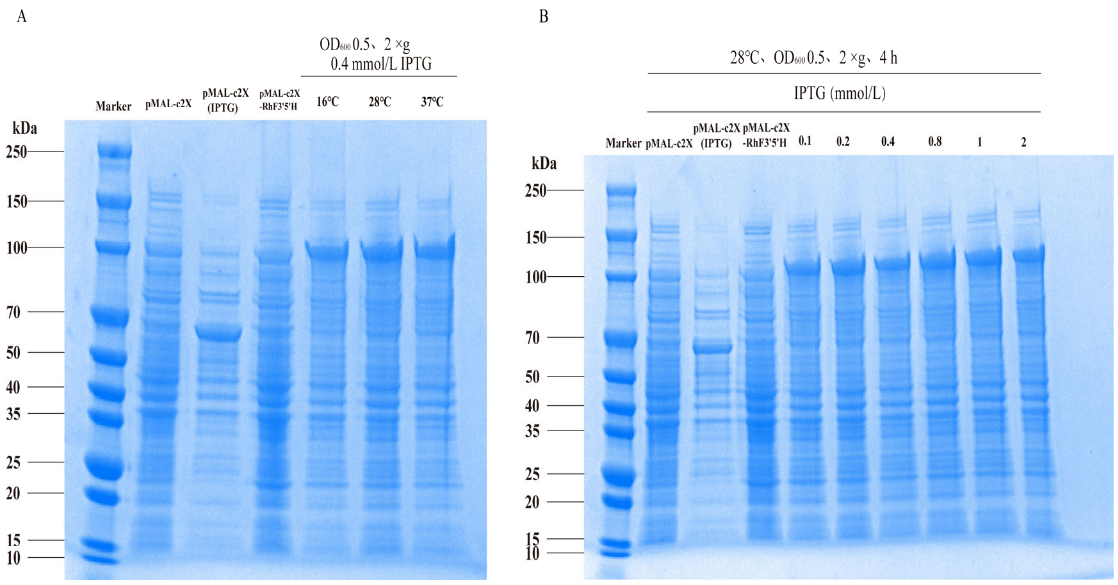

Supplementary Figure S6

Molecular Docking of naringenin and dihydrokaempferol with RhF3′5′H and Overexpression of the Gene with Anthocyanin Quantification. A: 3D cartoon representation of small molecule-target interactions. B: 2D interactions between the compound and its target.

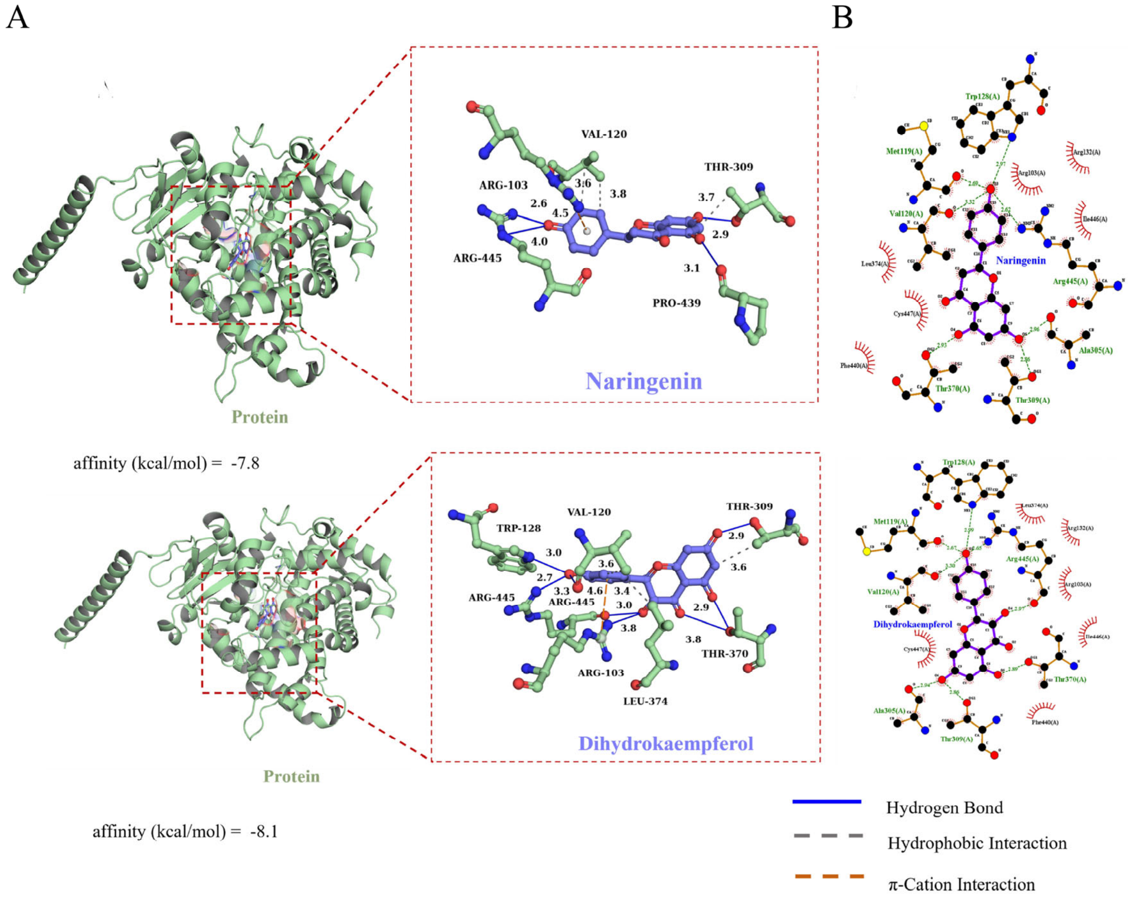

Supplement: Supplementary file 1 [file plants-15-01472-s001.zip › Supplementary figure .pdf]
