# Supplementary material for: Genome-Wide Identification of CYP75 Gene Family in Rhododendron simsii and Functional Analysis of Its Role in Promoting Anthocyanin Biosynthesis
Source: Plants (Basel). 2026 May 12;15(10):1472. doi: 10.3390/plants15101472 (PMC13210423; doi:10.3390/plants15101472)
Supplement: Supplementary file 1 [file plants-15-01472-s001.zip › Supplementary Table.pdf]

Table S1

The sequences of primers used in this study.

Primer name	Primer sequences (5'--3')	Purpose
cRhF3'5'H-F	ATGGCCGTAGACACTCTGTTGTT	cDNA Cloning
cRhF3'5'H-R	CTACATAGCATAAGCACTTGGTTGC	cDNA Cloning
qRhF3'5'H-F	AGACATGGTTTTCGCCGACT	qPCR
qRhF3'5'H-R	CCATGGCGAACGTCAACATC	qPCR
qActin-F	CACTGGTGTCATGGTTGGGA	Reference gene
qActin-R	CTCTTCAGGAGCAACACGGA	Reference gene
oRhF3'5'H-F	ACGGGGGACTCTTGACCATGGATGGCCGTAGACACTCTGTT	Overexpression
oRhF3'5'H-R	TACTAGTCAGATCTACCATGGCATAGCATAAGCACTTGGTT	Overexpression
pRhF3'5'H-F	GAATTCGGATCCTCTAGAATGGCCGTAGACAC	Prokaryotic expression
pRhF3'5'H-R	CAAGCTTGCCTGCAGGTCGACCATAGCATAAGCACTTG	Prokaryotic expression
qRhCYP75A1-F	TAGGTTGCACCCACCAGTTC	qPCR
qRhCYP75A1-R	ATAGGGTCCTCCCACACCTC	qPCR
qRhCYP75A2-F	ACTGGCCGAAATGATGCTGA	qPCR
qRhCYP75A2-R	GGCACGTCGGACTCTTGTAA	qPCR
qRhCYP75A3-F	ACATGGCCTACAATCGCCAA	qPCR
qRhCYP75A3-R	CCCCGTCTCTATAGCTCGGA	qPCR
qRhCYP75A$_4$-F	GAATCTATTCGCCGCCGGTA	qPCR
qRhCYP75A$_4$-R	GTTTCGGAACGTCGGACTCT	qPCR
qRhCYP75A$_5$-F	GGTCTGTTCAACGTAGGCGA	qPCR
qRhCYP75A$_5$-R	TCCCACTTGCTATGCATCCG	qPCR
qRhCYP75B$_1$-F	CGAGAGCTGCGAAATCAACG	qPCR
qRhCYP75B$_1$-R	TCCAAGGGATTGACCCATGC	qPCR
qRhCYP75B$_2$-F	GGTCTGTTCAACGTAGGCGA	qPCR
qRhCYP75B$_2$-R	TCCCACTTGCTATGCATCCG	qPCR
qRhCHS-F	GGTGCGAGATTGGTATTTGGA	qPCR
qRhCHS-R	TTGAACTCCCAAAGCCATTGT	qPCR
qRhF3H-F	GCGATGGCGGTACTCAGCATAC	qPCR
qRhF3H-R	GCTGGCGTGGATGGACTTGC	qPCR
qRhDFR-F	CCGTCAATGTTCAAGAGCACCAAC	qPCR
qRhDFR-R	GACACGAAGTACATCCAGCCAGTC	qPCR
qRhANS-F	AGCAACGGGAAGTACAAGAGCATC	qPCR
qRhANS-F	CAGAAAGCCGCCCACGAGATC	qPCR
vRhF3'5'H-F	TAAGGTTACCGAATTCCTTGACATGGTGGTGGCCGC	VIGS
vRhF3'5'H-R	GCTCGGTACCGGATCCCTCCGCCACCACCACCGC	VIGS

Table S2

Screening results of CYP75 gene family members in *Rhododendron simsii* genome

Sequence ID	Number of Amino Acid	Molecular Weight	Theoretical pI	Instability Inde	Aliphatic Index	Grand Average of Hydropathicity (GRAVY)
RsCYP75A2	512	56763.18	8.97	36.94	91.07	-0.025
RsCYP75A5	309	33833.82	9.26	36.99	96.63	0.135
RsCYP75A4	353	38773.24	9.44	37.62	89.04	-0.059
RsCYP75A3	335	37428.89	7.01	37.75	94.09	0.005
RsCYP75A1	659	72528.44	6	44.25	89.8	-0.017
RsCYP75B1	303	33345.79	9.55	29.96	98.88	-0.033

Table S3

Petal color classification and CIELAB color parameters of *Rhododendron* × *hybridum* under different experimental treatments.

This table summarizes the petal color classification based on the Royal Horticultural Society Colour Chart (RHSCC) together with the corresponding CIELAB color parameters of *Rhododendron* × *hybridum* petals. The evaluated groups included the red cultivar, pink cultivar, transient overexpression control (35S: GFP), *RhF3'5'H* overexpression treatment (35S-*RhF3'5'H*), VIGS control (pTRV2), and *RhF3'5'H* silencing treatment (pTRV2-*RhF3'5'H*). For each group, three biological replicates were measured. In the CIELAB color system, **L*** indicates lightness, **a*** indicates the red–green axis, **b*** indicates the yellow–blue axis, **C*** indicates chroma, and **H*** indicates hue angle. These data provide quantitative support for the phenotypic differences in petal coloration observed among cultivars and treatments.

Group	Number	RHSCC	CIELAB				
			L^*	a^*	b^*	C^*	H^*
Red	1	52A	3.81	11.85	-2.29	12.07	-0.19
	2	52A	4.32	10.53	-3.59	11.13	-0.33
	3	52A	4.94	11.46	-4.03	12.15	-0.34
Pink	4	68B	5.59	6.70	0.39	6.71	0.058
	5	68B	7.54	7.90	0.54	7.92	0.068
	6	68B	6.54	6.65	1.45	6.81	0.21
35S: GFP	7	52A	3.23	8.67	-2.61	9.054	-0.24
	8	52A	4.5	10.23	-3.34	10.76	-0.32
	9	52A	4.7	9.9	-0.8	9.93	-0.081
35S-RhF3′5′H	10	N57C	1.61	19.37	-0.28	19.37	-0.014
	11	N57C	1.66	14.77	-0.08	14.77	-0.0054
	12	N57C	1.55	14.06	-7.85	16.10	-0.51
pTRV2	13	51A	3.05	8.98	-3.32	9.57	-0.35
	14	51A	3.18	8.71	-0.23	8.71	-0.026
	15	51A	4.16	8.70	-1.29	8.80	-0.15
pTRV2- RhF3′5′H	16	68B	5.59	6.70	0.39	6.71	0.058
	17	68B	7.54	7.90	0.54	7.92	0.068
	18	68B	6.54	6.65	1.45	6.81	0.21
